# TIMP3 Regulates Mammary Epithelial Apoptosis with Immune Cell Recruitment Through Differential TNF Dependence

**DOI:** 10.1371/journal.pone.0026718

**Published:** 2011-10-28

**Authors:** Carlo V. Hojilla, Hartland W. Jackson, Rama Khokha

**Affiliations:** Department of Medical Biophysics, Ontario Cancer Institute, University of Toronto, Toronto, Ontario, Canada; Baylor College of Medicine, United States of America

## Abstract

Post-lactation mammary involution is a homeostatic process requiring epithelial apoptosis and clearance. Given that the deficiency of the extracellular metalloproteinase inhibitor TIMP3 impacts epithelial apoptosis and heightens inflammatory response, we investigated whether TIMP3 regulates these distinct processes during the phases of mammary gland involution in the mouse. Here we show that TIMP3 deficiency leads to TNF dysregulation, earlier caspase activation and onset of mitochondrial apoptosis. This accelerated first phase of involution includes faster loss of initiating signals (STAT3 activation; TGFβ3) concurrent with immediate luminal deconstruction through E-cadherin fragmentation. Epithelial apoptosis is followed by accelerated adipogenesis and a greater macrophage and T-cell infiltration in *Timp3* ^−*/*−^ involuting glands. Crossing in *Tnf* deficiency abrogates caspase 3 activation, but heightens macrophage and T-cell influx into *Timp3* ^−*/*−^ glands. The data indicate that TIMP3 differentially impacts apoptosis and inflammatory cell influx, based on involvement of TNF, during the process of mammary involution. An understanding of the molecular factors and wound healing microenvironment of the postpartum mammary gland may have implications for understanding pregnancy-associated breast cancer risk.

## Introduction

Tissue involution is a postnatal process that offers a unique window to study physiological cell death. Involution occurs in the thymus [1], prostate [2], uterus and mammary gland [3]. These tissues are subject to physiological cues that instruct them to undergo apoptosis to maintain cell number and homeostasis. In humans, the mammary gland undergoes extensive involution after the cessation of lactation [4], and this process has been widely studied in murine models. Post-lactational mammary gland involution serves to remodel the highly structured secretory gland into one that resembles the virgin state so that the differentiation program initiated by gestation may begin anew [5], [6]. Mammary involution depends on epithelial apoptosis in which epithelial lumens collapse and lobulo-alveolar structures are deleted with rapid elimination of up to 90% of the epithelium [7]. This process occurs in parallel with adipogenesis [8], and microarray expression analyses have suggested the involvement of inflammatory processes in mammary gland involution [9], [10]. Yet, factors that control these systems in parallel remain to be elucidated.

Mammary gland involution in the mouse has been described as a two-step process defined by the reversibility of involution [11], [12]. The first stage is marked by increased pro-apoptotic factors and can be reversed by re-induction of suckling. Death receptor-mediated and mitochondrial apoptosis signaling pathways are employed by mammary epithelial cells during involution. TNF and p55-TNFRI mRNA transcript levels decrease, while p75-TNFRII levels increase in the involuting mammary glands of rats [13]. Fas and FasL levels in glandular epithelia increase immediately upon pup withdrawal. As such, Fas-deficient *lpr* mice and FasL-deficient *gld* mice do not show apoptotic cells until the later stages of involution [14]. Specifically, TGFβ3, STAT3 and their downstream targets have been identified as initiators of the first phase of involution [15], [16], [17]. Mammary gland involution is also associated with cytochrome c release from the mitochondria, as well as the processing of initiator (caspases 8, 9) and executioner caspases (caspases 3, 7) which generally peak at 3 days after cessation of suckling in wild-type mice [14], [18].

The second irreversible phase of involution occurs around day 3 and involves widespread apoptosis and tissue remodeling, with extracellular matrix turnover and structural reorganization of the gland [19]. Matrix metalloproteinases (MMPs) and their tissue inhibitors (TIMPs) regulate matrix turnover during pubertal morphogenesis [20], [21], gestation, lactation [22], and involution [23], [24], [25]. While the first phase of involution is considered to be protease-independent [11], the second protease-dependent stage has decreased levels of TIMP and activated MMPs which initiate the deconstruction of the surrounding stroma therefore facilitating gland remodeling. At the same time, MMP activity has also been proposed to influence adipogenesis [8]. Synchronization of multiple compartments is essential for maintaining the functional integrity of the mammary gland during the phases of involution.

Interestingly, microarray and histological studies have revealed that inflammatory mediators are present during mammary gland involution [9], [10]. These arrays suggest the death receptor family involvement in the first phase of involution and the involvement of monocyte and lymphoid cytokines during the second phase [26]. Macrophages in particular have been found in the involuting glands of several species, and respond to the chemotactic signals provided by the remodeling matrix [27]. The involuting mammary gland microenvironment has been likened to wound healing and cancer environments [28]. Macrophages are known to play a role in involution, the wound healing response, and are also implicated in promoting breast cancer metastasis [27], [29]. Given that pregnancy associated breast cancers (PABC) have a poor patient outcome [30], [31], [32], a better definition of molecular factors that differentially regulate apoptosis from inflammatory cell recruitment will enhance our understanding of the processes underscoring physiological mammary involution and mammary cancer progression.

Both gain- and loss-of-function studies underscore the complex apoptotic role of TIMP3 [33]. TIMP3 is a negative regulator of inflammation in specific tissues [34], [35], [36] and in systemic responses to endotoxin [37]. TIMP3 deficient mice have accelerated post-lactation involution and fail to reinitiate lactation after 48hrs [23]. In this study, we ask whether TIMP3 functions to integrate the distinct programs of apoptosis and inflammation during homeostatic remodeling of post-lactational mammary involution. We identify the molecular and cellular events underlying the accelerated mammary gland involution in *Timp3* ^−*/*−^ mice. TIMP3 deficiency leads to a loss of the survival signal provided by E-cadherin-mediated cell to cell contact as well as an accelerated first phase of involution. A far greater and sustained influx of macrophages also occurs, which is coupled with an infiltration of T-cells in *Timp3* ^−*/*−^ involuting glands. Combining the TIMP3 and TNF deficiencies, we further dissect the TNF dependency of these molecular and cellular events. Our results show that TIMP3 is a critical physiological regulator for both apoptosis and inflammatory cell recruitment during murine mammary gland involution.

## Results

### Higher TNF levels and accelerated apoptosis during mammary involution in Timp3β mice

**Figure 1:**
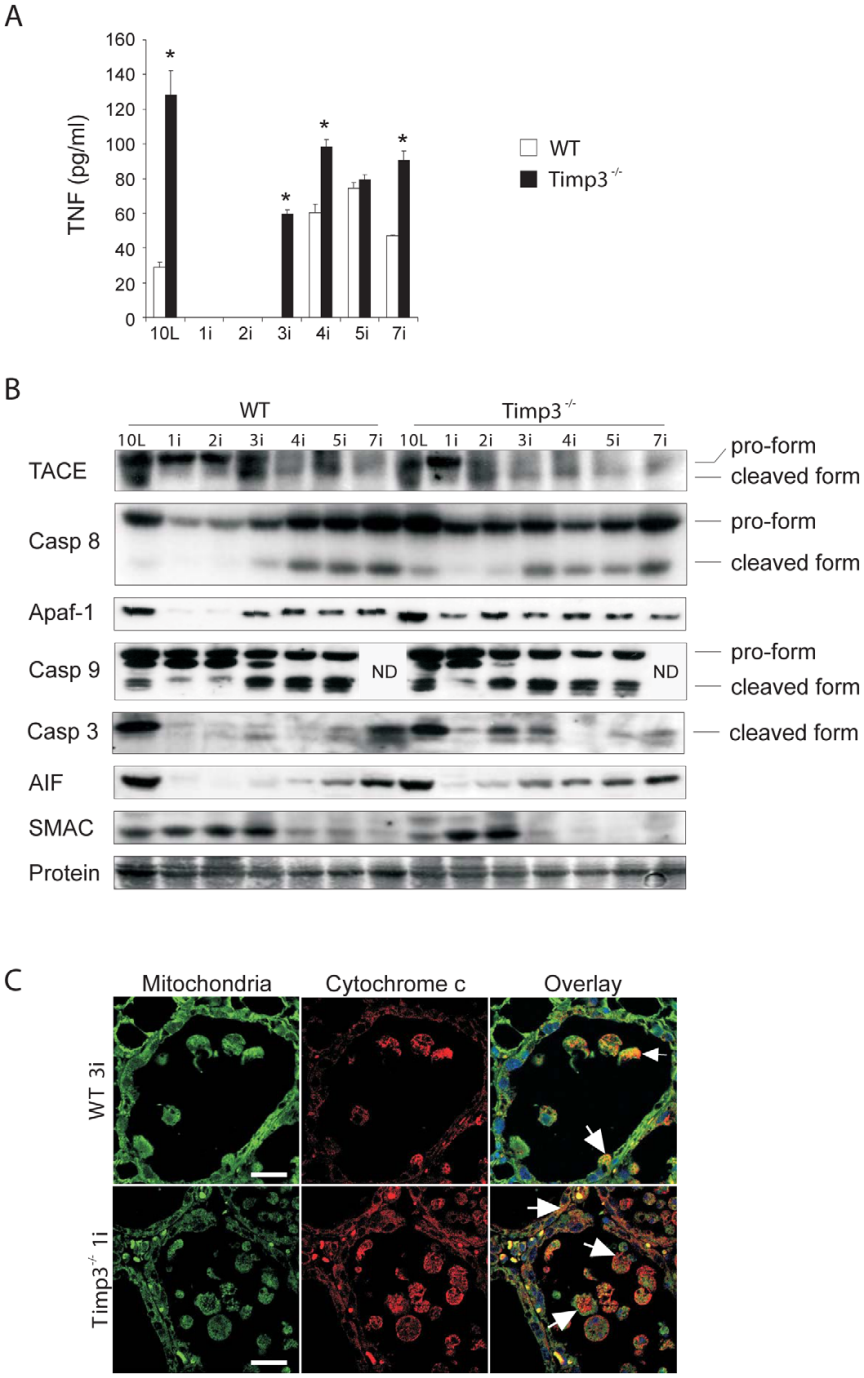
Increased TNF levels and apoptotic signaling in *Timp3*β involuting glands. (*A*) TNF levels as assessed by ELISA at different involution timepoints starting from day 10L until day 7i revealed higher levels of TNF levels in *Timp3* ^−*/*−^ glands (n = 3) compared to wild-type (WT, n = 3) both prior to the onset of involution and as an earlier rebound. (*B*) Representative western blot analyses of lysates from different involution timepoints probed for caspase 8 (Casp 8), Apaf-1, cytochrome c (Cyt c), caspase 9 (Casp 9; top band represents pro-caspase 9, bottom band represents cleaved caspase 9; ND = not determined for 7i), cleaved caspase 3 (Casp 3), Apoptosis-inducing factor (AIF), and Second mitochondria-derived activator of caspase (SMAC). (*C*) Immunofluorescence and co-localization of mitochondria (MitoTracker) and cytochrome c revealed earlier release of cytochrome c from the mitochondria (red signal, arrows) in *Timp3* ^−*/*−^ luminal cells, as well as cells that have detached and were shed into the lumen by 1i, than in wild-type at 3i. Bar graphs are expressed as mean ± SEM (* = *P*<0.02). Scale bar = 30 µm.

TIMP3 is the only TIMP capable of inhibiting the TNF alpha converting enzyme (TACE/ADAM17) [38], [39]. *Timp3* ^−*/*−^ mice have increased TNF levels and signaling owing to increased TACE activity in several systems [34], [35], [37]. We measured mammary TNF levels, which were detectable at the end of lactation, and again on day 4 of involution (4i) in wild-type mice. *Timp3* ^−*/*−^ mammary gland also displayed two distinct periods of TNF increase. It was 4-fold higher than wild-type at 10L, reappeared earlier and stayed higher than in wild-type tissue (Fig. 1A). Fas and FasL levels did not differ between wild-type and *Timp3* ^−*/*−^ glands throughout involution (data not shown). Death receptor-mediated apoptosis induced by TNF occurs through activation of the initiator caspase, caspase 8 [40]. Consistent with the elevated TNF levels in *Timp3* ^−*/*−^ glands, the cleaved form of caspase 8 was detectable at 10L and elevated earlier than wild-type (Fig. 1B).

We next examined the executioner arm of mitochondrial apoptosis, a multiprotein complex known as the apoptosome, which is comprised of cytochrome c, Apaf-1, and procaspase 9 [41], [42], [43]. These components showed an earlier temporal shift upon the loss of TIMP3 (Fig. 1B). Notably, Apaf-1 was present on 1i and 2i coinciding with the timing of maximal epithelial apoptosis in *Timp3* ^−*/*−^ mammary glands (Fata et al. 2001), but absent in wild-type tissue. Caspases 3 and 9 were also clearly activated earlier in TIMP3 null tissue as indicated by the presence of their cleaved forms (Fig. 1B). The loss of mitochondrial membrane integrity results in the release of cytochrome c into the cytoplasm [44]. Immunofluorescence showed mitochondrial cytochrome c release within luminal epithelial cells at 1i in *Timp3* ^−*/*−^ glands, but only at 3i in wild-type (Fig. 1C, arrows). The number of epithelial cells shed into lumens (Fig. 1C, arrowheads) with cytochrome c release was present in greater numbers in *Timp3* ^−*/*−^ glands compared to wild-type at 1i. We also observed apoptosis-inducing factor (AIF) expression to be earlier than wild-type in *Timp3* ^−*/*−^ involuting glands (Fig. 1B). The levels of second mitochondria-derived activator of caspase (SMAC), a mitochondrial-derived inhibitor of IAPs (inhibitors of apoptosis) [45], decreased sooner in *Timp3* ^−*/*−^ involuting glands (Fig. 1B). Thus, an earlier progression to apoptosis was indicated by the rapid appearance of several caspases and the loss of mitochondrial integrity in *Timp3* ^−*/*−^ involuting mammary glands.

### Mapping of first phase of involution and adipogenic signals

**Figure 2:**
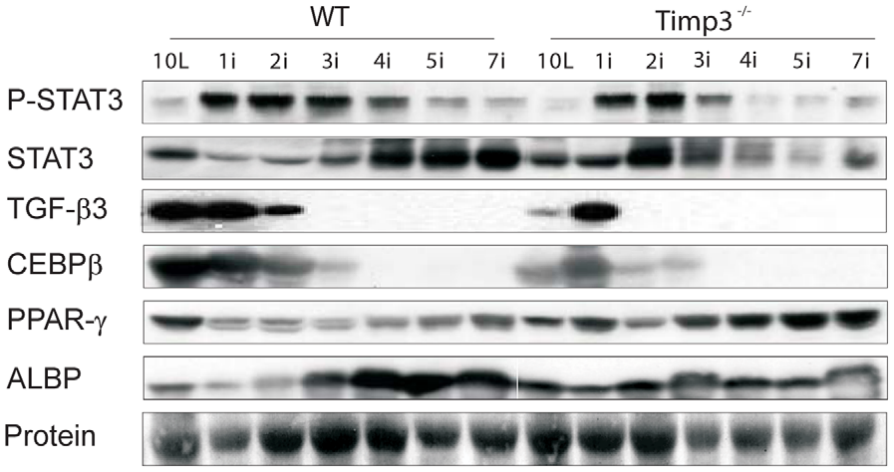
Mapping of priming and adipogenic signals in *Timp3* ^−^^*/*−^ mammary involution. Representative western blot analyses of lysates from wild-type (WT) and *Timp3* ^−*/*−^ mammary glands at different involution timepoints starting from day 10L until day 7i probed for phosphorylated STAT3 (P-STAT3), total STAT3, TGF-β3, and the adipogenic markers: CEBPβ, PPAR-γ, and ALBP.

Murine mammary gland involution occurs in two phases where involution is reversible in the first 48 hours. *Timp3* null mice fail to re-establish lactation after 48 hours of involution [23]. STAT3 is known to be involved in this early phase and STAT3 loss, or loss of its upstream activators, abrogates the first phase and delays the onset of involution [17], [46], [47]. We observed that total STAT3 was upregulated earlier in *Timp3* ^−*/*−^ mammary gland. STAT3 was phosphorylated immediately upon induction of involution in both wild-type and *Timp3* ^−*/*−^ mammary glands, but activation of STAT3 was turned off earlier in *Timp3*β and not seen past 3i (Fig. 2). Milk stasis during involution also rapidly induces TGFβ-3 levels in the mammary gland to induce apoptosis [15]. We observed that *Tgfβ-3* expression lasted until 2i in wild-type glands, but was undetectable after 1i in *Timp3* ^−*/*−^ glands (Fig. 2) consistent with the rapid completion of the first phase of involution.

In mammary involution, adipogenesis is important for the re-establishment of the pre-pregnant state filling out the space left behind by epithelial apoptosis [8]. We therefore examined markers of adipocyte differentiation: CEBP-β for early [48] and PPAR-γ for late adipocyte differentiation, and ALBP as a marker for adipocytes [49]. Generally, an earlier and increased levels of PPARγ and ALBP coincident with decreased CEBPβ levels indicated faster onset of the adipogenic program (Fig. 2).

### E-cadherin breakdown compromises epithelial architecture in Timp3^−/−^ mammary glands

**Figure 3:**
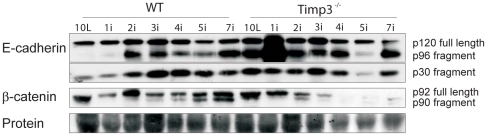
Fragmentation of E-cadherin and disruption of adherens junctions. (*A*) Representative western blot analyses of lysates from wild-type (WT) and *Timp3* ^−*/*−^ mammary glands at different involution timepoints starting from day 10 of lactation (10L) until day 7 of involution (7i) probed for E-cadherin and β-catenin, showing early processing of E-cadhern and β-catenin in *Timp3* ^−*/*−^ mammary glands.

We determined whether TIMP3 loss affected E-cadherin-mediated cell-cell contact since this adhesion molecule is processed by MMPs [50], [51], and we earlier reported an aberrant MMP2 activation during mammary involution in *Timp3* ^−*/*−^ mice [23]. E-cadherin breakdown was evident by 2i in wild-type mammary gland. On the other hand, an immediate and far greater fragmentation of E-cadherin occurred at 1i in *Timp3* ^−*/*−^ mammary tissue (Fig. 3), which was even present at day 10 of lactation (10L), suggesting that this tissue may have compromised epithelial cell-cell contact during lactation. Also notable was the overall elevated level of E-cadherin in *Timp3* ^−*/*−^ glands. There was a corresponding disappearance of β-catenin by 4i in *Timp3* ^−*/*−^ involuting glands (Fig. 3) in contrast to wild-type tissue, suggesting the earlier destabilization of E-cadherin-mediated adherens junctions consistent with compromised cell-cell contact in this tissue. Altogether the data illustrated in Figures 1, 2, and 3 provide a map of key molecular signals involved in physiological apoptosis and adipogenesis in the mammary gland, where TIMP3 deficiency leads to an earlier deconstruction and remodeling of the epithelial compartment.

### Re-establishment of survival signaling by recombinant TIMP3

**Figure 4:**
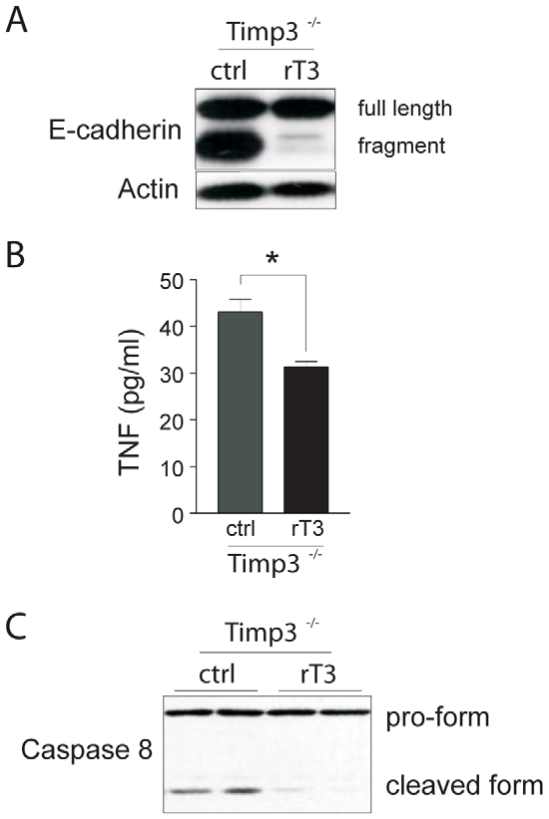
Inhibition of E-cadherin fragmentation and TNF signaling by recombinant TIMP3. Pellets containing vehicle control (ctrl) or recombinant human TIMP3 (rT3) were implanted at the beginning of involution and tissues were harvested at day 3 of involution. (*A*) Pellets containing recombinant TIMP3 blocked E-cadherin fragmentation at 3i in *Timp3* ^−*/*−^ (n = 3). (*B*) The increase in TNF levels at 3i in *Timp3* ^−*/*−^ tissue glands was reversed by recombinant TIMP3 as measured by ELISA. (*C*) Caspase 8 activation at 3i was only abrogated in *Timp3* ^−*/*−^ regressed tissue that was reconstituted with recombinant TIMP3. Bar graphs are expressed as mean ± SEM (* = *P*<0.01; n = 3).

Next we asked whether TIMP3 plays a direct role in inhibiting E-cadherin breakdown and TNF bioactivity during mammary involution. TIMP3-containing slow-release Elvax pellets were implanted distal to the lymph node in the fourth inguinal mammary fat pad at the onset of involution and tissue immediate to the pellet was collected at 3i. Reconstitution of TIMP3 rescued E-cadherin fragmentation in *Timp3* ^−*/*−^ mice (Fig. 4A). The increased TNF level in *Timp3* ^−*/*−^ glands was also significantly reduced (*P*<0.01) by this manipulation (Fig. 4B). Furthermore, activation of caspase 8 was reduced in TIMP3-reconstituted null tissue (Fig. 4C). These data show that TIMP3 protein could rescue the alterations associated with accelerated mammary involution in *Timp3* ^−*/*−^ mice.

### Timp3 deficiency initiates inflammatory and immune cell recruitment in involuting glands

**Figure 5:**
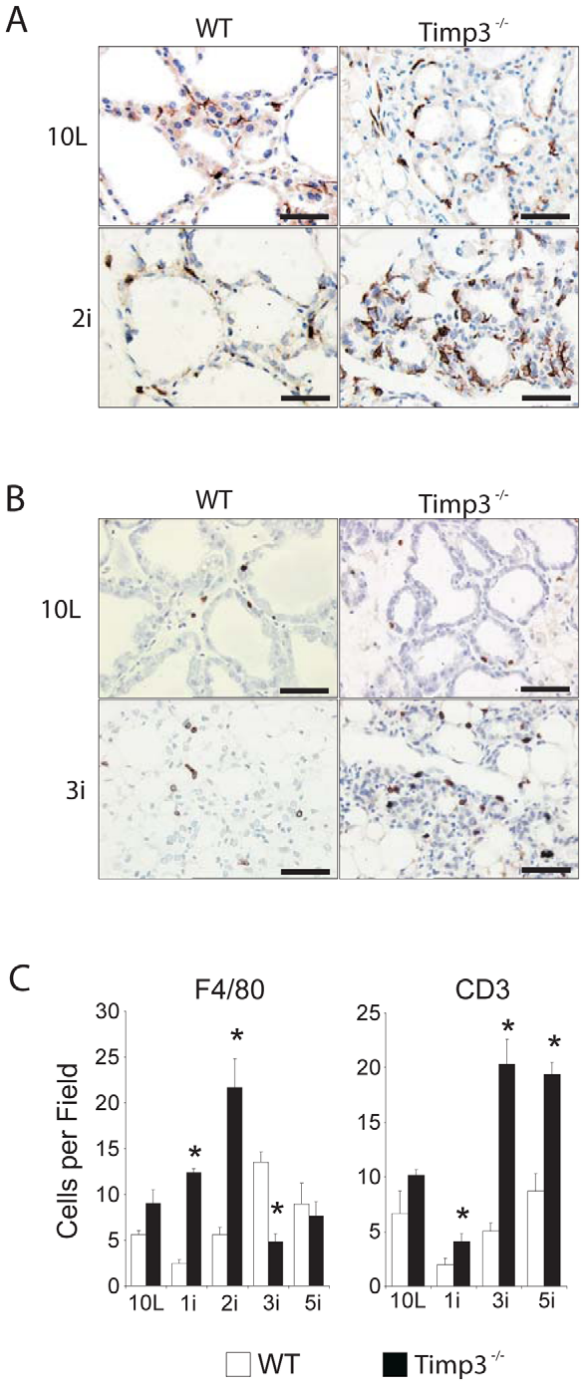
Increased and sustained inflammatory and immune response in *Timp3* ^−^^*/*−^ involuting glands. (*A*) Representative images of F4/80-positive cells during wild-type (WT) and *Timp3* ^−*/*−^ involution at day 10L and day 2i (*B*) Representative images of CD3^+^ cells during involution at 10L and 3i. (*C*) F4/80-postive and CD3-positive cell counts as assessed by histomorphometry. (*P* values for F4/80: are 0.01 for 1i 2i and 3i; *P* values for CD3: 1i, 0.04; 3i, 0.03; 5i, 0.02) for WT (n = 3) and *timp3* ^−*/*−^ glands (n = 3). Bar graphs are expressed as mean ± SEM. Scale bar = 30 βm.

Historically, studies in dairy animals (cow, sheep and goat) have described neutrophil and macrophage infiltration during the process of mammary gland involution [52], [53], [54]. Microarray studies in the mouse also support an inflammatory component to involution [9], [10]. Since we previously identified TIMP3 as a negative regulator of inflammation in several systems [34], [55], we assessed the infiltration of inflammatory cells into involuting mammary glands. Neutrophil influx occurred during involution and was comparable between wild-type and *Timp3* ^−*/*−^ involuting glands (data not shown). In contrast, macrophage numbers were comparable during lactation, but increased up to 4-fold (*P*<0.01) in *Timp3* ^−*/*−^ involuting glands at early stage involution (1i and 2i) when compared to wild-type glands, and rapidly declined thereafter at 3i (Fig. 5A). The maximal macrophage influx in wild-type glands was at 3i, however these did not reach levels seen in *Timp3* ^−*/*−^ glands. This represented an abrupt and immediate initiation of macrophage infiltration in mammary glands lacking TIMP3.

The presence of lymphocytes has been indicated in the involuting mammary glands of ruminants [56], [57], but has yet to be studied in the murine model. Given that lymphocytic infiltrates were found in aged *Timp3* ^−*/*−^ livers [34], we measured the infiltration of B and T-cells in involuting mammary glands. CD3-positive T-cells were present in alveolar and ductal epithelium, including interalveolar and periductal areas, at low levels in wild-type but were elevated in *timp3* ^−*/*−^ glands throughout involution. The number of T-cells in involuting *Timp3*-deficient glands peaked at 3i and was sustained until 5i. The wild-type tissue displayed similar kinetics but a smaller increase of these cells. The T-cell infiltration exhibited different kinetics compared to the macrophage influx, temporally occurring subsequent to macrophage (Fig. 5B). B cells were very sparse in the mammary gland and remained comparable between wild-type and *Timp3* ^−*/*−^ glands, as assessed by anti-B220 immunohistochemistry (data not shown).

### The role of TNF in mammary involution of the Timp3 deficient gland

**Figure 6:**
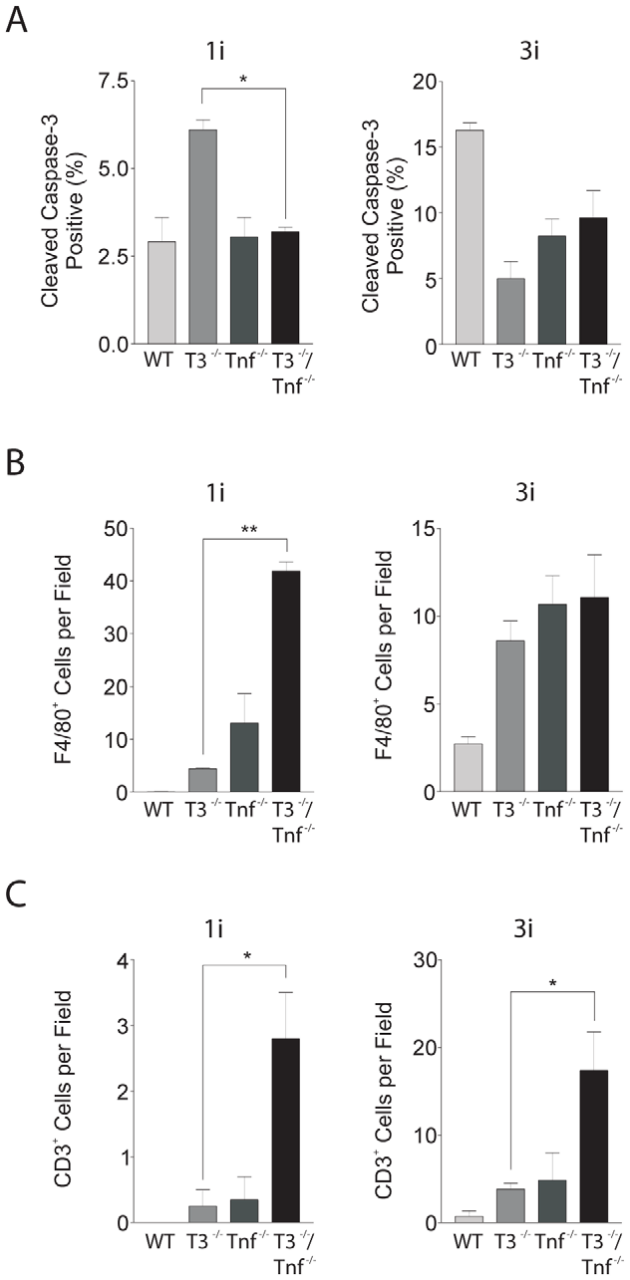
Dependence of TNF on caspase 3 activation, inflammatory and immune cell influx in involuting mammary glands. Histomorphometric analyses of wild-type (WT; n = 4 mice), *Timp3* ^−*/*−^ (*T3* ^−*/*−^; n = 4), *Tnf* ^−*/*−^ (n = 4), and *Timp3* ^−*/*−^ *Tnf* ^−*/*−^ (n = 4) involuting glands at days 1 and 3 of involution (1i and 3i): (A) cleaved caspase 3; (B) F4/80-positive cells; and (C) CD3-positive cells. Bar graphs are expressed as mean ± SEM. * *P*<0.05; ** *P*<0.01.

TIMP3 regulates the bioactivity of the inflammatory cytokine TNF since it is the physiological inhibitor of the TNF sheddase TACE. We have shown TIMP3 to be a critical regulator of physiological systems that depend on TNF such as liver regeneration and apoptosis [34], [58] and the innate immune response to endotoxins [36], [37]. We therefore asked whether increased apoptosis and inflammatory reaction during mammary involution in *Timp3* ^−*/*−^ mice was dependent on increased TNF bioactivity, by combining the *Timp3* and *Tnf* deficiencies. For these experiments we used *Timp3* mice that we backcrossed seven times into the C57BL/6 background to match the *Tnf* deficient mice, whereas the data in Figures 1, 2, 3, 4, and 5 were generated using pure FVB/N *Timp3* ^−*/*−^ mice. Activation of caspase 3 is a measure of apoptosis downstream of death receptor signaling by TNF, and its positivity has been previously reported in regressing mammary glands [59]. The percentage of mammary epithelial cells that stained positive for active caspase 3 as quantified by histomorphometry of single (*Timp3* ^−*/*−^ or *Tnf* ^−*/*−^) and compound (*Timp3* ^−*/*−^/*Tnf* ^−*/*−^) knockouts in the C57BL/6 background at 1i and 3i (Fig. 6A). The higher number of activated caspase 3-positive cells in *Timp3* ^−*/*−^ glands compared to wild-type confirmed greater apoptosis in C57BL/6 *Timp3* ^−*/*−^ mice at 1i similar to FVB/N *Timp3* ^−*/*−^ strain [23]. Also as expected, the apoptosis index was higher in wild-type at 3i compared to *Timp3* ^−*/*−^ mice, which coincide with the expected peak of epithelial apoptosis in involuting wild-type glands. Deletion of *Tnf* in *Timp3* null mice resulted in significantly reduced cleaved caspase 3 positive cells compared to *Timp3* ^−*/*−^ at 1i, showing that the lack of TNF rescued the accelerated apoptosis in *Timp3* ^−*/*−^ glands (*P*<0.05; Fig. 6A). A control group with TNF deletion alone also showed reduced numbers of epithelial cells with activated caspase 3 at 3i, also confirming the role of the TNF pathway during normal mammary involution.

We next examined whether the greater inflammatory cell influx seen during mammary involution of *Timp3* ^−*/*−^ mice depended on TNF (Fig. 5, 6B, C). Intriguingly at 1i, the number of F4/80-positive macrophages was increased by at least 4-fold in *Timp3* ^−*/*−^/*Tnf* ^−*/*−^ when compared to *Tnf* ^−*/*−^ glands, and up to 8-fold (*P*<0.01) when compared to *Timp3* ^−*/*−^ glands (Fig. 6B, 5C). At 3i, the overall numbers of macrophages subsided and became comparable between single and compound knockout groups, but still remained higher than that in wild-type glands (Fig. 6B). Examination of CD3-positive T-cells revealed a significant 3-fold increase (*P*<0.05) at 1i following compound removal of TIMP3 and TNF (Fig. 6C). This trend of increased T-cell recruitment was maintained at 3i in *Timp3* ^−*/*−^/*Tnf* ^−*/*−^ involuting glands (*P*<0.05; Fig. 5C). Contrary to our expectation the inflammatory and immune cell influx was not ameliorated by adding *Tnf* deficiency to *Timp3*-deficient mice. This series of experiments indicated that TNF is required for accelerated mammary epithelial apoptosis but not immune cell influx of *Timp3* null involuting glands.

## Discussion

**Figure 7:**
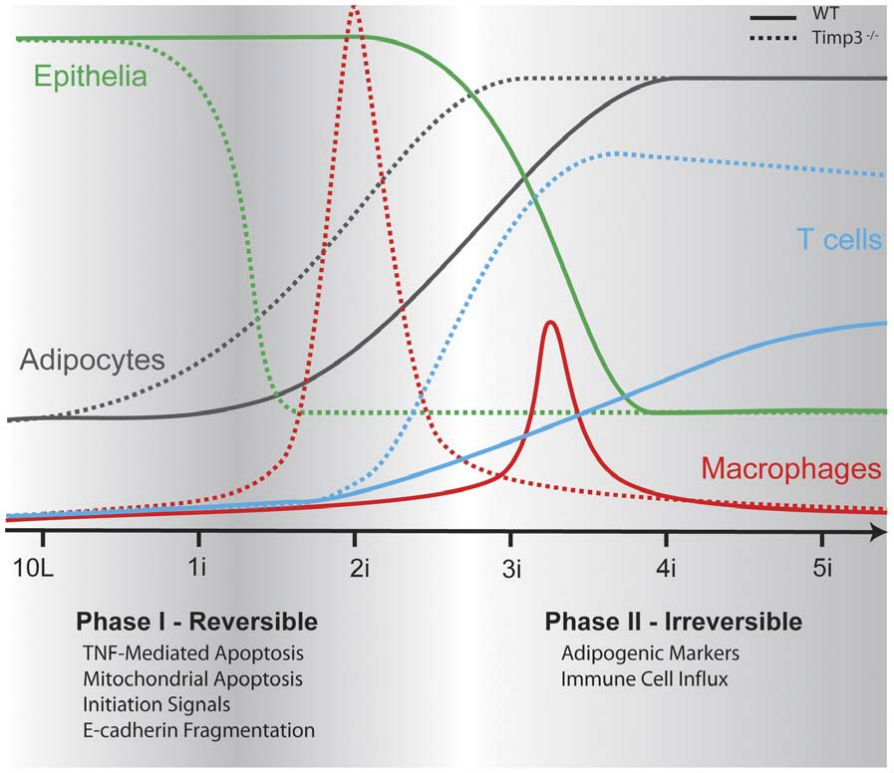
Schematic representation of the shifted timeline of involution in *Timp3* ^−^^*/*−^ mammary glands. The timing and intensity of epithelial apoptosis, adipogenesis, and immune cell influx are depicted by a solid line for wild-type and a dotted line for *Timp3* ^−*/*−^βmammary gland involution.

The rapid mammary involution established in the *Timp3* null mouse provides a model to study the different phases and cellular compartments of this remodeling tissue. We analyzed several key molecules essential for epithelial cell survival and explored inflammatory cells during mammary gland regression, as temporally mapped in Figure 7. The loss of the extracellular metalloproteinase inhibitor, TIMP3, accelerated the first phase of involution leading to an early onset of the irreversible second phase [23]. We observed extensive E-cadherin fragmentation and an immediate onset of TNF-induced apoptosis coinciding with pSTAT3 activation and TGFβ3 expression, which are recognized initiators of involution. The second phase was marked by early adipogenesis and prominent immune cell infiltration. The ability of TIMP3 to differentially impact specific aspects of inflammation that have been proposed during involution[26], such as death receptors and ligands during the first phase and cytokine/chemokines dependent immune cell influx during the second phase, is revealed upon its combination with TNF deficiency.

An early event in *Timp3* ^−*/*−^ mammary gland involution is E-cadherin fragmentation and the loss of β-catenin resulting in the loss of a critical survival signal that provides structural integrity. Targeted deletion of E-cadherin in the mammary gland leads to unscheduled apoptosis during pregnancy such that the gland resembles involuting tissue [60]. Vallorasi *et al.* have shown that the truncation of E-cadherin is present at the initiation of mammary involution [61]. Synthetic MMP inhibitors prevent E-cadherin processing [50], [62] and we have found increased MMP2 activation in a variety of *Timp3* deficient systems [35], [63], [64]. In particular, the immediate activation of MMP2 at the onset of mammary involution in *Timp3* deficient glands [23] coincides with E-cadherin fragmentation, which is mitigated by reconstitution with recombinant TIMP3. The presence of fragmentation products at 10L suggests that epithelial structures may even be compromised during lactation in *Timp3* null mice. We propose that the loss of E-cadherin triggers an impending change in mammary architecture that sensitizes to apoptosis. Beyond mammary gland involution, another in vivo model has demonstrated that the loss of TIMP3 sensitizes tissues to cell death pathways. During liver regeneration the loss of TIMP3 initiates an apoptosis program due to excessive TNF [34]. In this study, TNF dysregulation was concurrent with increased caspase 8 activation and accompanied by an accelerated timeline of mitochondrial apoptosis in *Timp3* ^−*/*−^ involuting mammary glands. The immediate peak of apoptosis in *Timp3* ^−*/*−^ glands was attenuated by the genetic ablation of TNF, thereby confirming the role of TNF in the first phase of involution.

The loss of TIMP3 created an environment with greater macrophage infiltration in comparison to wild-type mammary glands. It is likely that this inflammatory influx does not initiate apoptosis, because the peak of macrophage infiltration follows the peak of apoptosis in both wild-type and *Timp3* ^−*/*−^ glands. Apoptotic cell bodies must be removed by phagocytosis either by neighboring epithelial cells or by professional phagocytes such as macrophages in order to maintain tissue homeostasis during mammary involution [65], [66], [67], [68]. The number of CD3^+^ T-cells were also elevated and sustained in *Timp3* ^−*/*−^ glands following the influx of macrophages. To date, lymphocyte infiltration has only been described in bovine and ovine mammary gland involution [56] and their function in the mammary gland remains to be defined. We observed a pronounced macrophage and T-cell influx upon the compound loss of TIMP3 and TNF, indicating a TNF-independent mechanism for their recruitment into a TIMP3-deficient microenvironment. Compound TIMP3 and TNF deficiency in the heart also leads to a 5-fold greater neutrophil infiltration in an otherwise non-inflammatory system [35]. Since involution related inflammation has been theorized to play a role in PABC, this separation of epithelial apoptosis from inflammation provides insight into the mechanisms of inflammation during this critical period.

Our results highlight the pleiotropic ability of the extracellular metalloproteinase inhibitor TIMP3 during mammary gland involution. TIMP3 influences distinct processes through TNF-dependent and TNF-independent pathways with its loss accelerating and amplifying specific involution events. TIMP3 acts as a safeguard against untimely physiological cell death, unscheduled inflammatory response, and premature loss of mammary gland function. A greater understanding of apoptotic and inflammatory regulators provides insight into PABC-related risk factors.

## Materials and Methods

### Ethics Statement

This study was carried out in strict accordance with the recommendations in the Guide for the Care and Use of Laboratory Animals of the National Institutes of Health with all efforts made to minimize suffering. The protocol was approved by the University Health Network Animal Care Committee (Animal Use Protocol #812).

### Mice and Experimental Involution

For the majority of the analyses, wild-type and *Timp3* ^−*/*−^ mice were in FVB/N background. For the compound knockout studies with TNF, wild-type, *Timp3* ^−*/*−^, *Tnf* ^−*/*−^, and *Timp3* ^−*/*−^/*Tnf* ^−*/*−^ mice were all in C57/BL6 background. Forced experimental involution was performed as previously described [23] according to guidelines established by the Canadian Council for Animal Care under protocols approved by the Ontario Cancer Institute Animal Care Committee.

### Antibodies and Reagents

The antibodies used were: Apaf-1 (Upstate); caspase 9 and activated caspase 3 (Cell Signaling); E-cadherin, β-catenin, cytochrome c, SMAC, and Mitotracker CD3, F4/80, B220 (BD Biosciences); anti-neutrophil antibody (Serotec); AIF (gift from Dr. J. Penninger); caspase 8 (gift from Dr. R. Hakem). Recombinant human TIMP3 was prepared according to the manufacturer's specifications (R&D Systems).

### Protein collection, western blotting, and TNF ELISA

Mammary gland collection and lysate preparation were performed as previously described [23]. A total of 3 independent, non-sibling females were used for each timepoint per genotype. Protein quantification was performed using Dc Biorad Assay as per manufacturer's instructions. Equal loading was rigorously assessed and adjusted for, based on amido black staining of newly transferred gels and silver staining of SDS-PAGE gels that were electrophoresed in parallel. The amido black-stained gels and silver-stained gels were included and labeled as ‘Protein’ in the Figures. For western blotting experiments, 50 µg of total protein were loaded onto SDS-PAGE gels. Densitometry analyses were performed using ImageQuant and Northern Eclipse software. TNF ELISA was performed as previously described [34].

### Pellet implantation

50 µg of recombinant human TIMP3 (R&D Systems) were loaded onto pellets as per manufacturer's instructions. Implantation of pellets was done as previously described [23].

### Confocal microscopy and Immunocytochemistry

Two photon confocal microscopy was used for all immunofluorescence and immunocytochemistry analyses. Exposure time, gain, and offset parameters were first empirically determined and then kept constant throughout. Preparation of mammary glands for immunofluorescence and immunocytochemistry were performed as previously described [23]. Depending on the primary antibody used, control IgG antibodies and secondary antibodies alone were used for controls to assess specificity of primary antibodies.

### Immunohistochemistry and Histomorphometry

Preparation of mammary glands for immunohistochemistry were performed as previously described [23], using 1∶50 dilutions of anti-activated caspase 3 (Cell Signaling); anti-CD3, anti-F4/80, and anti-B220 (BD Pharmingen); and anti-neutrophil (Serotec). Histomorphometric counts of were performed on the fourth inguinal gland, using 10 random fields distal to the lymph node.

### Statistical Analyses

Statistical analyses were performed for observations that have at least three mice per group. Student's t test was performed for statistical analyses between two groups (WT and *Timp3* ^−*/*−^). One-way ANOVA was performed for statistical comparison between four groups (WT, *Timp3* ^−*/*−^, *Tnf* ^−*/*−^, and *Timp3* ^−*/*−^/*Tnf* ^−*/*−^), followed by Tukey test for pair-wise statistical analysis.
